# Effect of borax-modified activator on mechanical properties and drying shrinkage of alkali-activated slag/metakaolin mortar

**DOI:** 10.1038/s41598-024-58172-x

**Published:** 2024-04-08

**Authors:** Haiming Chen, Ziguang Qin, Jie Chen, Yadong Zhang, Peng Wu

**Affiliations:** 1 School of Civil Engineering and Architecture, Anhui University of Science and Technology, Huainan, 232001 China; 2 Engineering Research Center of Underground Mine Construction, Ministry of Education, Anhui University of Science and Technology, Huainan, 232001 China

**Keywords:** Civil engineering, Composites

## Abstract

Alkali-activated materials (AAMs) possess several advantages, such as high strengths and low carbon emissions. However, their application is hindered due to their significant shrinkage. This study explored the effect of borax-modified sodium silicate activator and metakaolin (MK) on the mechanical properties and drying shrinkage (DS) of alkali-activated slag (AAS) and AAS/MK (AASM) mortars. X-ray diffraction, scanning electron microscopy, and Fourier-transform infrared spectroscopy were used to characterize the hydration products. The results showed that the DS reduction of the AAS mortar was related to decreased Na_2_O content, a reduction in the proportion of mesopores, and the formation of moisture-retaining borate compounds. The DS reduction of the AASM mortar was attributed to the ultra-fine differential effect induced by MK, reducing the connected pores. The modified activator combined with MK increased the chemically bound water content in the matrix. Additionally, the B–O bond and highly active MK improved compactness of the AASM mortar. The use of borax-modified activators and MK provides a new solution to address the significant shrinkage issue in AAMs. This sets the stage for AAMs to potentially replace OPC, contributing to low-carbon emissions and promoting environmental protection.

## Introduction

The cement industry emits approximately 1.45 Gt of CO_2_ per year, accounting for approximately 4% of global fossil fuel emissions^1,2^. As infrastructure construction continues to expand in developing countries, greater use of ordinary Portland cement (OPC) is expected, resulting in more pronounced environmental pollution from cement production. Alkali-activated materials (AAMs) offer an environmentally friendly solution for reducing carbon emissions in the cement industry. These materials primarily use industrial solid waste (e.g., slag^3–5^, lithium slag^6,7^, and fly ash^8,9^) as a low-carbon binder for precursors^10^. This approach not only provides cost-effective benefits but also promotes solid waste reuse, leading to a reduction in CO_2_ emissions of approximately 40%^11^. Previous studies have demonstrated that AAMs exhibit high strengths, low permeabilities, and excellent corrosion resistances, making them promising alternatives to cement-based materials^12,13^. However, the issue of drying shrinkage (DS) poses a significant obstacle to the application of AAMs. Cracks resulting from DS accelerate the penetration of harmful substances such as carbon dioxide, acid, and base ions, thereby reducing the materials' durability. Consequently, reducing the shrinkage is a theoretically effective approach to enhance the erosion resistance of AAMs.

AAMs condense quickly, and DS primarily occurs in the initial stage of hydration and the subsequent gradual evaporation of free water during curing. The widely recognized mechanism of DS is that when the water gradually evaporates, surface tension is generated when the menisci are formed in the gel pores and capillary pores, thereby causing an equal compressive stress in the skeleton and causing volume shrinkage^14,15^. Current research primarily focuses on reducing the DS of AAMs by incorporating fibers^16–18^, chemical admixtures^4,19^, and mineral admixtures^20,21^, as well as changing the curing system^22–24^. Changing the curing system, while effectively reducing shrinkage, may also lead to a decrease in mechanical properties and issues of unstable internal curing humidity^24^. Adding fibers can enhance the toughness of AAMs and reduce shrinkage, but it also introduces uncertain factors such as uneven fiber distribution and lower fluidity than cement mortar^25^. Research has indicated that the replacement of 10–20% slag with metakaolin (MK) can lead to a reduction in the autogenous shrinkage of AAMs by approximately 40–50%^26,27^. Additionally, Asaad et al.^20^ discovered that substituting MK for slag enhanced the durability of AAMs, manifesting in reduced DS and increased resistance to aggressive environmental conditions. The activator brings greater alkalinity to AAMs than OPC, which is one of the key factors affecting DS. As a commonly used activator in AAMs, liquid sodium silicate accelerates the polycondensation reaction of the system due to the presence of [SiO_2_(OH)_2_]^2−^, which has a better excitation effect but also results in rapid setting and greater shrinkage to AAMs^11,28^. Conversely, NaOH and Na_2_CO_3_ as activators exhibit lower shrinkage than Na_2_SiO_3_ but compromise the mechanical properties of AAMs^29,30^. The choice of activators significantly affects the performances of AAMs. Therefore, by modifying the types of ions present in the activators, there is a possibility of improving the performances of AAMs.

Borate ions have been shown to act as retarders in cement-based materials^31,32^. Compounds containing borate ions, such as borax and boric acid, have been successfully used as retarders in various cement types, such as Portland cement and magnesium phosphate cement^33,34^. The retarding mechanism involves the reaction between calcium ions and borate ions to form hydrated calcium borate. This compound covers the surfaces of clinker particles, partially or fully, thereby decelerating clinker dissolution and postponing hydration^32^. Some studies^8,35,36^ have investigated the curing of borate ions in AAMs and found that they can enhance the workability of AAMs. Rakhimova et al.^36^ prepared activators containing borate ions with pH values of 8.5 and 10.5, simulating radioactive solutions containing borates in pressurized water reactors of nuclear power plants. The results showed that both solutions resulted in alkali-activated slag (AAS) paste with satisfactory setting times and adequate compressive strengths at 28 d, utilizing an alkali equivalent of 7%. Boron (B) shares similar coordination characteristics with silicon (Si) and aluminum (Al). Theoretically, [BO_4_] will likely share oxygen atoms with [AlO_4_] and [SiO_4_] to change the gel structure, thus influencing the macro- and micro-properties of AAMs.

Recently, some studies have attempted to partially replace commonly used activators with borates in AAMs and geopolymers, obtaining satisfactory outcomes in both mechanical performance and workability^37–39^. Revathi et al.^37^ investigated the performance changes of fly-ash-based geopolymers by partially substituting sodium silicate with borax. Substituting 10–30% of the sodium silicate resulted in a final setting time of over 200 min for the geopolymer without affecting the mechanical properties at 28 d. In addition, tetrahedral boron absorption bands with wavenumbers of 1380–1310 cm^−1^ and 1134 cm^−1^ were observed via Fourier-transform infrared spectroscopy (FTIR). Attenuated total reflectance (ATR) FTIR showed that B partially replaced the Al in the gel network, indicating that Si–O–T(B, Al) was compatible with the gel structure. Bagheri et al.^39^ compared the environmentally friendly substitution of borax in AAMs and geopolymers and found that a 10–30% borax content improved the mechanical properties of the geopolymers more significantly than AAMs. Li et al.^40^ investigated the impact of borax as an admixture on the performance of AAS mortar. Their research revealed that, despite the initial formation of calcium borate complexes due to borax addition, which slowed down the hydration reaction, it had no effect on the later-stage strength development of the AAS mortar. Moreover, borax was found to prolong setting time in most low-calcium AAMs. In fly ash-based AAMs, a linear increase in setting time was observed the dosage of borax increased from 2 to 8%^38^. Sajjad et al.^41^ studied the influence of borax as a retarder on the properties of one-part alkali-activated fly ash/slag binders. The research suggested that borax effectively prevented flash setting in AAMs, but exceeding a 4% dosage reduced their mechanical performance.

While these studies provided detailed micro-level and workability analyses, research on the shrinkage performances and durability of AAMs in this new excitation environment is still limited. If the addition of borax leads to excessive shrinkage, the application value of AAMs may be diminished. In general, in calcium-rich AAS systems and calcium-poor geopolymers, [SiO_4_]^4−^ and [AlO_4_]^5−^ produced by different activators dissolve the precursor materials and balance with alkali metal cations (Ca^2+^, Na^+^) to form C–S–H and C(N)–A–S–H^42^. However, under different excitation environments, the polycondensation reaction in AAMs may change, resulting in variations in the reaction speed or the resulting gel network and subsequent performance changes. For instance, the presence of certain concentrations of [BO_3_]^3−^, [BO_4_]^5−^, and [PO_4_]^3−^ in the activator has the potential to alter the alkalinity of the mortar's pore solution and the internal humidity of AAMs^39,43^.

Based on environmental sustainability and durability considerations, this study explored the influence of a boron environment on the DS and mechanical properties of different AAMs using a modified sodium silicate activator with borax. Experimental investigations were conducted to analyze the factors of the borax content on the fluidity, DS, mechanical properties, and microscopic characteristics of the AAS mortar to determine the optimal borax content. With the optimal borax content, the impact of various metakaolin (MK) contents (5%, 10%, 15%, and 20%) as an alternative to ground granulated blast furnace slag (GGBS) in the AAS mortar was studied. The results demonstrated that the boron environment reduced the DS and improved the mechanical properties of AAMs. Additionally, substituting MK for slag further decreased the DS while enhancing the mechanical properties of AAMs. Therefore, borax-modified activators offer a method to improve the performances of AAMs, contributing to carbon emissions reductions and environmental protection.

## Materials and methods

### Materials

**Figure 1:**
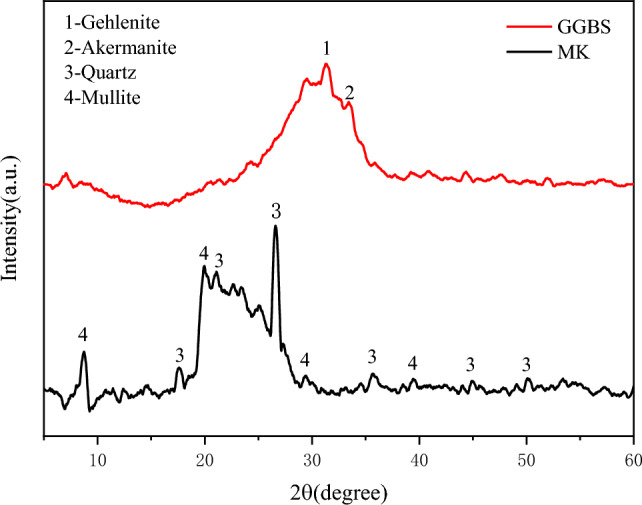
GGBS and MK raw materials’ XRD patterns.

**Figure 2:**
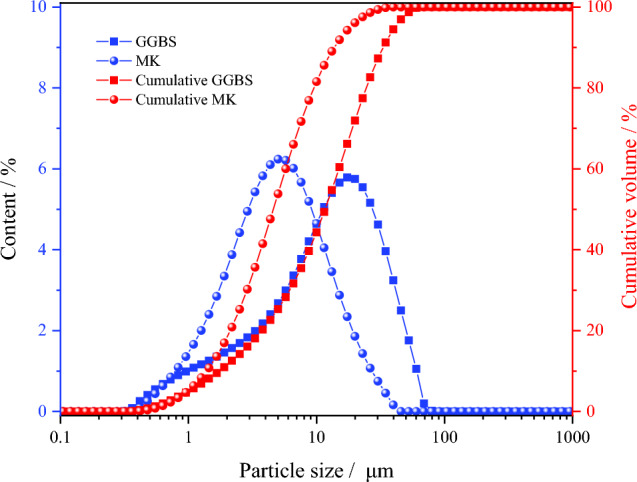
Particle size distribution of GGBS and MK.

S95 GGBS and MK were sourced from Gongyi Wanying Environmental Protection Materials Co., Ltd. (China), and their chemical compositions and X-ray diffraction (XRD) patterns are presented in Table 1 and Fig. 1, respectively. The particle size distributions of the GGBS and MK were measured using a laser particle size analyzer, as shown in Fig. 2, indicating median particle sizes of approximately 17.38 and 5.01 μm for GGBS and MK, respectively. Liquid sodium silicate was obtained from Guangzhou Suixin Chemical Co., Ltd. (China), with an initial modulus (Ms) = 3.57, where Ms is the ratio of the amount of SiO_2_ to the amount of Na_2_O. The main indicators are provided in Table 2. The content of liquid sodium silicate with different Ms was adjusted with an appropriate proportion of sodium hydroxide and deionized water to obtain different Ms Values. The sand used in this study was sourced from the Huaihe River in China, with a fineness modulus of 2.36 (Type II) and an apparent density of 2550 kg/m^3^. Anhydrous borax (Na_2_B_4_O_7_), which was obtained from Tianjin Guangfu Development Co., Ltd. (China), is a white powder, and its aqueous solutions are weakly alkaline.

**Table 1:** Chemical composition (%) of GGBS and MK measured by XRF.

Composition	SiO_2_	Al_2_O_3_	CaO	MgO	Fe_2_O_3_	TiO_2_	SO_3_	Na_2_O	MnO	K_2_O	Others
GGBS	31.81	18.44	34.59	9.93	0.29	1.01	2.45	0.62	0.30	0.40	0.16
MK	48.91	49.55	-	0.06	0.76	0.13	0.02	0.04	0.01	0.48	0.04

**Table 2:** Physical and chemical indicators of liquid sodium silicate.

Modulus	Baume degree (°Bé)	Na_2_O (wt%)	SiO_2_ (wt%)	Density (g/cm^3^, 20 °C)
3.57	39.7	8.32	28.77	1.38

### Mix proportions

In this study, nine experimental groups were established to analyze the effects of modified activators on the performances of different AAMs. According to previous studies^20,36^, the water–binder ratios for all groups were maintained at 0.41, the cement–sand ratio was 0.5, the adjusted Ms was set at 1.2, and the water in the activator was calculated as part of the mixing water. The alkali equivalent levels tested were 4.9%, 5.6%, 6.3%, and 7%. The activator substitution levels examined were 10%, 20%, and 30%. Additionally, the GGBS replacement levels investigated were 5%, 10%, 15%, and 20%. Table 3 shows the mixed design of AAS and AASM mortars. The control groups, C-N7 and C-N5.6, did not contain borax and MK. C-N5.6 represents the AAS mortar with an alkali equivalent of 5.6%. The B-M groups were slag/MK systems, such as B20-M10, where B20 indicates a replacement of 20% of the sodium silicate with borax, and M10 represents a replacement of 10% of the slag with MK. Na_2_O content (alkali equivalent) indicates the percentage of Na_2_O mass in the activator to the total mass of the cementitious material.

**Table 3:** Mix proportions of mortars (g/kg).

Mix ID	Na_2_O content (%)	Borax to Na_2_SiO_3_ (wt%)	Materials consumption(g/kg)			
			GGBS	MK	Na_2_SiO_3_	Borax
C-N7	7	0	685	0	315	0
C-N5.6	5.6	0	732	0	268	0
B10-M0	6.3	10%	685	0	283.5	31.5
B20-M0	5.6	20%	685	0	252	63
B20-M5	5.6	20%	650.75	34.25	252	63
B20-M10	5.6	20%	616.5	68.5	252	63
B20-M15	5.6	20%	582.25	102.75	252	63
B20-M20	5.6	20%	548	137	252	63
B30-M0	4.9	30%	685	0	220.5	94.5

### Methods

#### Fluidity tests

The fluidity of the fresh mortar was assessed following ASTM C1437-20^44^. The mortar was poured into a truncated cone mold and compacted uniformly by ramming the rod from the periphery toward the center. Excess mortar above the mold's top surface was removed using a trowel. Subsequently, the mold was gently lifted, and the flow table test was immediately initiated. The table was vibrated once per second, completing 25 vibrations in approximately 25 ± 1 s. After vibration, the maximum spread diameter and orthogonal length of the mortar on the flow table were measured using a steel ruler, and the average value was calculated.

#### Flexural and compressive strengths tests

The flexural and compressive strengths of the specimens were determined according to ASTM C348-21^45^ and ASTM C349-18^46^ respectively. The raw materials were mixed intensively for 3 min using a mortar mixer to ensure uniformity. Subsequently, water and activator were added and stirred for 2 min. Fresh mortar was poured into a polyethylene mold (40 mm × 40 mm × 160 mm) and vibrated on a vibration table for 5–10 s to eliminate air bubbles. The specimens were placed in a curing room maintained at a temperature of 23 ± 2 °C and a humidity of above 95%. After 1 d of curing, the specimens were placed in a curing box under the same conditions after demolding and cured to 3, 7, and 28 d. The flexural and compressive strengths were calculated using Formulas (1) and (2) respectively^47^:

1 $$\sigma_{f} = { 2}.{8}P$$

2 $$\sigma_{c} = \, 0.{62}P$$

where *σ*_*f*_ is the flexural strength, MPa; *σ*_*c*_ is the compressive strength, MPa; and *P* is the maximum load in the strength test, kN.

#### Drying shrinkage tests

DS amounts of all the specimens were tested following ASTM C596-18^48^. Three columnar specimens (25 mm × 25 mm × 280 mm) were prepared for each group, and the molds were removed after 24 h of curing in the curing room. The specimens were submerged in water at a temperature of 20 ± 1 °C for 48 h and then removed. The initial length was measured using a comparator after wiping the samples with a wet cloth. The measuring accuracy of the comparator is 0.001 mm. Subsequently, the samples were stored indoors at a temperature of 20 ± 1 °C and a humidity of 50% ± 3%. The length changes will be measured during the curing process until a specified age period. The microstrain was calculated according to Formula (3):

3 $$\mu_{\varepsilon } = \frac{{L_{0} - L_{T} }}{280} \times 10^{6}$$

where *μ*_*Ɛ*_ is the microstrain; *L*_0_ is the initial length of the specimen, mm; *L*_*T*_ is the test length of the specimen at the age to be tested, mm.

#### Mercury intrusion porosimetry tests

The pore structure characteristics of the mortar samples were tested using mercury intrusion porosimeter (MIP). The mercury pressure range was 0.1 to 61,000 psia, and the contact angle was set to 130°.

#### Microscopic tests

The specified aged paste and mortar samples were immersed in anhydrous ethanol for 7 d and then dried in a vacuum chamber for 48 h. The microstructures of the mortar samples were analyzed using scanning electron microscopy (SEM, Flex 1000). Before the analysis, a thin layer of gold was sputtered onto the test block using an MSP-2S magnetron ion diffractometer for 90 s to enhance the conductivity. The samples used for the SEM observations were slices taken from the selected mortars after the compressive test was completed. Characterization of hydration products using X-ray diffractometer (XRD, Smartlab SE) manufactured in Japan and Fourier-transform infrared spectroscopy (FTIR, Nicolet IS50). The XRD analysis was carried out using a BD68000162-01 X-ray diffractometer with CuKα radiation at 40 kV and 50 mA. The scanning range was established between 5° and 60°. The samples were scanned at a rate of 5°/min with an interval of 0.01° to collect the data. FTIR testing scanned 32 times from 4000 to 400 cm^−1^ at 4 cm^−1^ resolution.

## Results and discussion

### Fluidity

**Figure 3:**
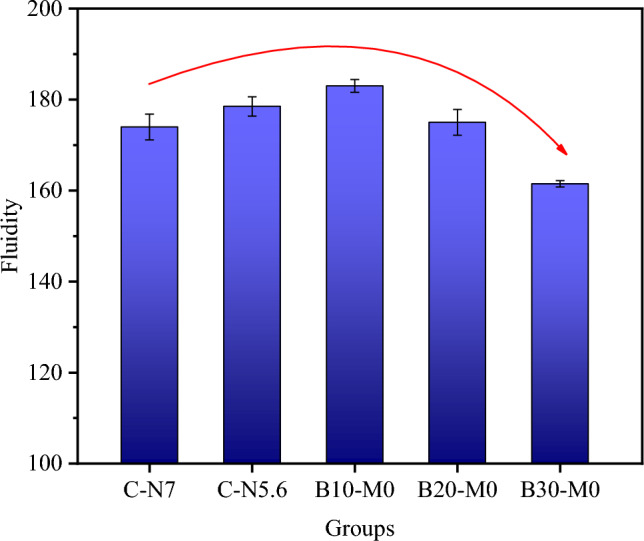
Fluidity of AAS mortars with different borax content in the modified activator.

Figure 3 shows the effect of the borax-modified activator on the fluidity of the AAS mortar: with an increasing borax content in the sodium silicate activator, the mortar's fluidity initially increased and then decreased. Among the samples, B10-M0 achieved the highest fluidity at 183 mm, which represents a 5% increase over the fluidity of C-N7 at 174 mm. However, an excess of borax led to a decrease in the mortar's fluidity, with B30-M0 displaying the lowest fluidity at 161.5 mm, marking a 7.18% reduction. In comparison to C-N7, C-N5.6 exhibited a fluidity of 178.5 mm, signifying a 2.52% enhancement, suggesting that reducing alkali equivalent contributed to the increase of the mortar's fluidity. The fluidity change of the AAS mortar was primarily influenced by two factors: (1) Alkali equivalent. Borax partially substituted the activator, thereby reducing its Na_2_O content. The positive impact of the Na_2_O content on the fluidity of the AAS mortar was attributed to the introduction of additional [SiO_4_]^4−^ by the activator, which enhanced the electrostatic repulsion between particles and improved the dispersion of free water. This led to a decrease in the apparent viscosity of the AAS mortar and a consequent increase in its fluidity^49^. Figure 3 shows that the lower the substitution rate, the higher the alkali equivalent and fluidity of the mortar. An excessive amount of Na_2_O in the activator accelerated the dissolution and polycondensation reactions of gel particles, resulting in decreased fluidity.^50^. Therefore, C-N7 exhibited lower fluidity than C-N5.6. (2) Free water content. The dissolution of borax gradually consumed free water. In comparison to the effect of electrostatic repulsion, the limited availability of free water for dispersing GGBS particles made the fluidity more sensitive to the loss of free water^51,52^. Borax hydrolysis produced boric acid and borate ions under alkaline conditions^40^. The hydrolysis reaction equations for borax are shown in (4) and (5).

4 $$(\text B_{4} \text O_{7} )^{2 - } + 7 \text H_{2} \text O \to 2 \text {OH}^{ - } + 4 \text H_{3} \text {BO}_{3}$$

5 $${\text H}_{3} \text {BO}_{3} + \text {OH}^{ - } \to \text B(\text {OH})_{4}^{ - }$$

**Figure 4:**
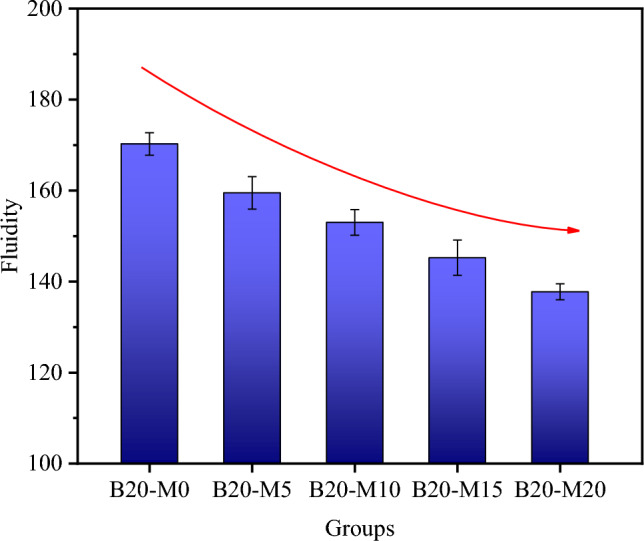
Fluidity of the AASM mortar with different MK content.

Figure 4 shows the fluidity of the fresh AAS/MK (AASM) mortar. Incorporating MK decreased the fluidity of the mortar, and the greater the amount of MK added was, the lower the fluidity was. The fluidity of B20-M0 was 170.25 mm. With the increase in the dosage of MK, the fluidity of the AASM mortar across all groups decreased by 6.31%, 10.13%, 14.68%, and 19.09%, respectively. The reduced fluidity of the AASM mortar was primarily attributed to the smaller particle size and higher water absorption of MK compared to GGBS. The substitution of MK for a portion of GGBS increased the density of the AASM mortar, resulting in a decrease in its fluidity. Simultaneously, the higher water-absorbing capacity of MK led to the early loss of free water in the AASM mortar, further impacting its fluidity^53^.

### Flexural and compressive strengths

**Figure 5:**
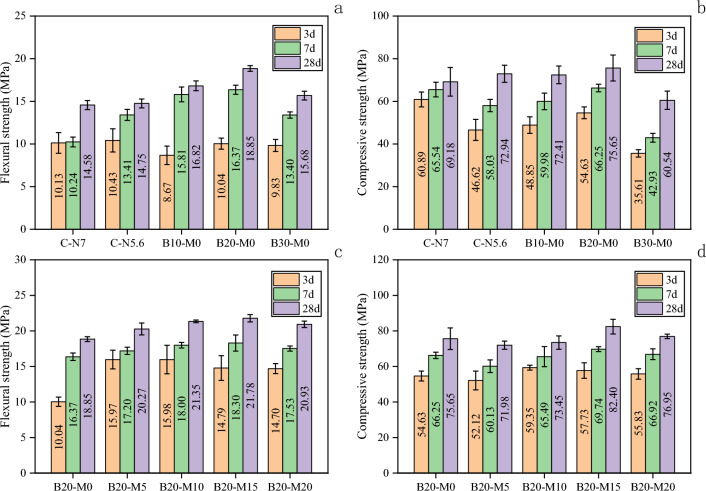
Flexural and compressive strengths of the AAS and AASM mortars.

An appropriate proportion of the borax-modified activator proved beneficial in optimizing the strength of the AAS mortar. As shown in Fig. 5a, with the increase in the borax substitution rate, the flexural strength (FS) of the AAS mortar exhibited an initial increase followed by a subsequent decrease. Figure 5b presents a consistent trend in the compressive strength (CS) of the AAS mortar, similar to FS. The FS of the AAS mortar at both 7 d and 28 d exceeded that of the control group. The optimal substitution level was found to be 20%, and the CS and FS at 28 d of B20-M0 were respectively 9.35% and 29.29% higher than those of C-N7 in the control group. The 7-d CS of C-N5.6 was 11.46% lower than that of C-N7, while the FS and CS were similar at 28 d. Additionally, the addition of borax decreased the strength of the AAS mortar at 3 d. However, when borax was introduced under the same alkali equivalent conditions, the optimized modified AAS mortar (B20-M0) exhibited higher FS and CS at both 7 d and 28 d compared to C-N5.6 and C-N7. This clearly illustrated the positive impact of incorporating borax on the development of strength in the AAS mortar.

When sodium silicate is used as the sole activator, the alkali equivalent is crucial to the strength development of the AAS mortar. A higher alkali equivalent facilitated the dissolution of precursor materials such as GGBS more effectively than a lower one^54–56^. However, exceeding a certain alkali equivalent range, the activator would rapidly dissolve GGBS in the early stage, accelerate the polymerization reaction of [SiO_4_], [AlO_4_] and Ca^2+^, causing excessive gelation. This hindered later hydration reactions, ultimately reducing the mechanical properties. The incorporation of the borax-modified sodium silicate activator mitigates these issues by reducing rapid condensation associated with high alkali equivalent. In the initial stages of hydration, borate ions formed calcium borate complexes with Ca^2+^, which adhered to unreacted GGBS and slowed down the condensation process. Contrary to gelatinization, this complex reaction mainly affected the AAS mortar strength at the 3 d due to the semipermeable membrane properties of the complex on GGBS surface. As hydration progressed, the complex gradually permeated and disintegrated, normalizing the hydration reaction^40^. The complex reaction was shown in formula (6). Moreover, [BO_4_]—generated from borax hydrolysis and sharing similar coordination characteristics with [SiO_4_] and [AlO_4_]—enhance gel polymerization^37^. Maintaining an appropriate proportion of borax is essential, as a significant reduction in sodium silicate content would reduce the activator's alkalinity, weakening its ability to dissolve GGBS and affecting hydration reactions.

6 $${2 \text B(\text {OH})}_{4}^{ - } + \text {Ca}^{2 + } \to \text {Ca}(\text {B}(\text {OH})_{4} )_{2}$$

Figure 5c shows that the FS of the AASM mortar increased initially and then decreased with the increase in the MK content. The optimal substitution rate was determined to be 15%. The FS of B20-M15 increased by 15.54% at 28 d compared with that of B20-M0. As shown in Fig. 5d, with the increase in the MK content, the CS of the AASM mortar showed a trend of decreasing first, then increasing, and then decreasing. The 28-d CS of B20-M15 increased by 8.92% compared with that of B20-M0, which was 12.97% higher than that of C-N5.6.

The factors contributing to the enhanced mechanical properties of the AASM mortar with MK can be summarized as follows. As the substitution rate of MK increased, the concentrations of alumina (Al_2_O_3_) and silica (SiO_2_) in the mortar also rose, which promoted the geopolymerization reaction and the formation of N–A–S–H and C–A–S–H gels^20^, ultimately enhancing mechanical properties. Additionally, MK possesses a finer particle size and a larger specific surface area compared to GGBS. Its high reactivity facilitated the hydration reactions, thereby improving the interfacial bonding performance and compactness of the AASM mortar. However, when the MK content exceeded 20%, there was a reduction in the calcium oxide from GGBS, which lowered the proportion of C–(A)–S–H gel and consequently diminished the mechanical properties of the AASM mortar.

### Porosity

**Figure 6:**
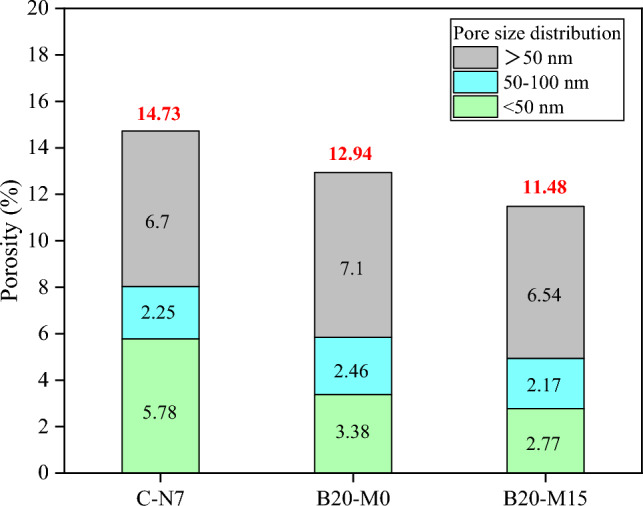
Pore size classification of AAS and AASM mortars.

**Figure 7:**
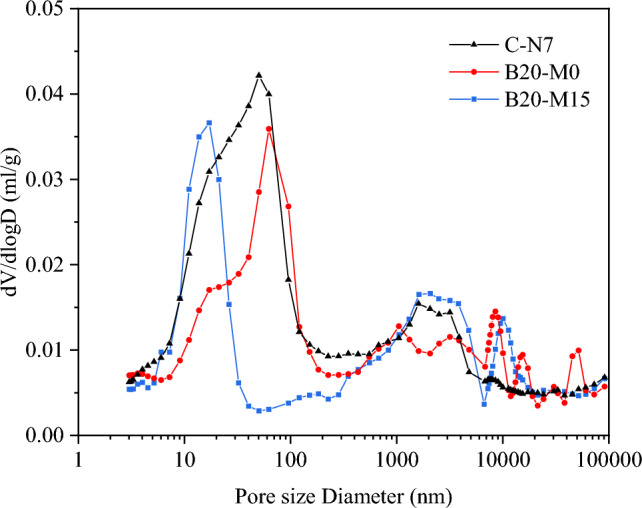
Pore size distribution of AAS and AASM mortars.

According to the International Union of Pure and Applied Chemistry (IUPAC) definition, porous materials are categorized into three classes based on pore size: micropores (< 2 nm), mesopores (2–50 nm), and macropores (> 50 nm)^21^. In order to analyze the impact of borax-modified sodium silicate activator and MK on the porosity of the AAS and AASM mortars, a comparison of the pore size distribution was conducted among the initial control group (C-N7), the optimal group B20-M0, and B20-M15. Figure 6 illustrates the pore size distribution of the AAS and AASM mortars, revealing recorded porosities at 28 d of 14.73%, 12.94%, and 11.48% for the three mortar samples, respectively. The proportions of pores smaller than 50 nm were 5.78%, 3.38%, and 2.77%, respectively. In general, the modified mortar exhibited decreased porosity and a reduced proportion of mesopores. The characteristics of the pore diameter distribution of the mortars were shown in Fig. 7, with all three curves showing a peak value, corresponding to the most probable pore diameter, representing the pore size with the highest frequency within different pores. The most probable pore diameter for the control group and B20-M0 are around 50 nm, while for B20-M15, it was around 20 nm. Overall, the proportion of pores in the modified mortars smaller than 50 nm was lower. The reduced porosity in the AAS and AASM mortars primarily attributed to the [BO_4_] generated during borax hydrolysis. These ions participated more actively in the polymerization process compared to when only sodium silicate was used as the activator, enhancing formation of C–(A)–S–H gel and refining the mortar's pore structure. The inclusion of MK further reduced by promoting geopolymerization, which led to the additional formation of N–A–S–H gel and a denser matrix. With its finer particle size relative to GGBS, MK also contributed to pores filling. The variation in porosity was directly related to mortar’s strength, showing that lower porosity translated to higher.

### Drying shrinkage

**Figure 8:**
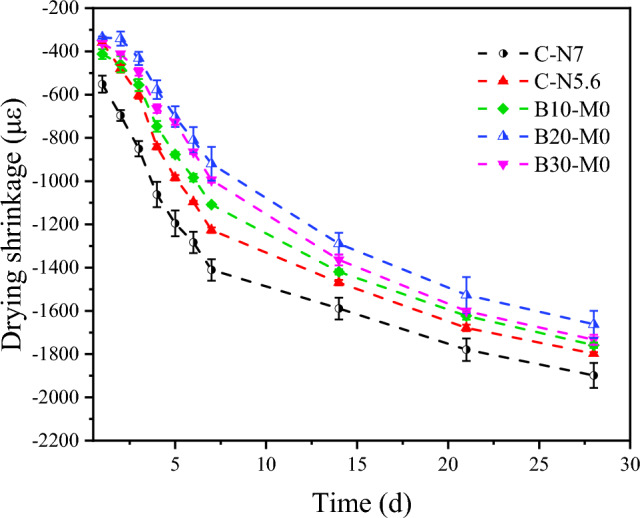
DS of mortars with different borax content in the modified activator.

When AAMs are exposed to acid, base ions, and CO_2_, the cracks resulting from DS can exacerbate the extent of the damage. The DS value serves as a measure of the volume change in an AAM caused by water evaporation, allowing for the evaluation of the susceptibility of AAMs to damage. In Fig. 8, the DS values of C-N5.6 at 7 d and 28 d were recorded as 1226.79 με and 1798.21 με, respectively, whereas for C-N7, the values were 1410.71 με and 1898.81 με, respectively. The DS value for C-N7 at 28 d was 5.30% higher than that of C-N5.6, suggesting that an increase in alkali equivalent led to a rise in the DS of the AAS mortar. For B10-M0, B20-M0, and B30-M0, the DS values at 7 d were 1108.93 με, 919.64 με, and 994.64 με, respectively, and at 28 d, they measured 1758.93 με, 1662.5 με, and 1733.93 με, respectively. The DS values for the modified mortar initially decreased and then increased as the content of borax was increased. B20-M0 demonstrated the optimal performance, showing a 34.81% and 12.45% reduction in the DS values at 7 d and 28 d, respectively, compared to C-N7.

According to the capillary tension theory, in an unsaturated air environment, the loss of free water in cementitious materials generates surface tension within gel pores and capillaries, leading to isotropic compressive stresses and consequent volume shrinkage. Collins et al.^14^ pointed out that significant shrinkage stresses are primarily associated with mesopores smaller than 50 nm, while macropores (> 50 nm) and micropores (< 2.5 nm) remain unaffected. MIP analysis of B20-M0 and B20-M15 revealed a decreased proportion of mesopores, indicating an improved with the composite activator, which mitigated the mortar's DS. When sodium silicate was used alone as the activator, an increase in the alkali equivalent accelerated the hydration reaction, leading to increased gel pores and resulting in greater DS^57^. In line with previous studies, a higher alkali equivalent accelerates condensation, adversely affecting the mortar's volume stability^21^. When using a borax-modified activator, the reduction in the dosage of the sodium silicate activator was beneficial for reducing the DS. Borate compounds formed contributed to moisture retention, stabilizing the internal humidity and further reducing DS^51^. However, excessive borax substitution, which reduced alkali equivalent to 4.9%, hindered the later development of the gel network and weakened the matrix's resistance to shrinkage stresses. It is recommended to maintain a borax substitution level of approximately 20% to optimize the AAS mortar performance.

**Figure 9:**
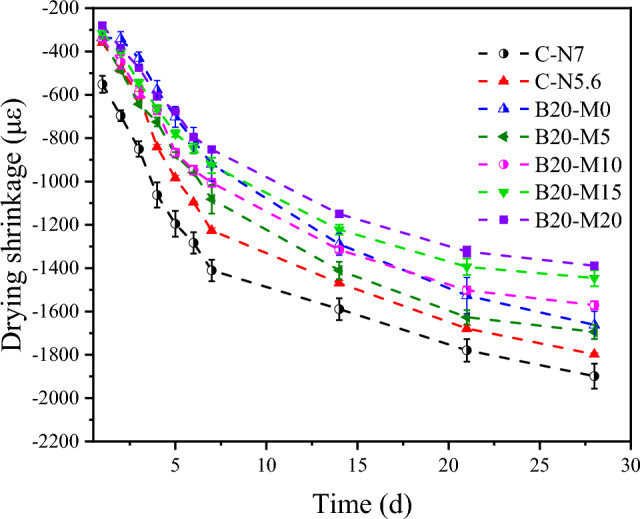
DS of mortars with various ratios of MK to GGBS.

Figure 9 illustrates the evolution of the DS values in the AASM mortar containing 5%, 10%, 15%, and 20% MK within a boron environment. The DS values at 28 d for B20-M5, B20-M10, B20-M15, and B20-M20 were 1694.64 με, 1571.43 με, 1446.43 με, and 1389.29 με, respectively. Except for B20-M5, each group exhibited a decrease in DS values compared to B20-M0 at 28 d, with reductions of 5.48%, 13.00%, and 16.43%, respectively. As the substitution rate of MK for GGBS increased, the mortar's DS values gradually decreased. The growth rate of the DS for the AASM mortar slowed down after 7 d in comparison to the AAS mortar, indicating that the incorporation of MK contributes to a further reduction in the DS values of the mortar.

The increase in MK content effectively reduced the DS of the AASM mortar for the following reasons. MK's finer particle size and higher specific surface area induced an ultrafine differentiation effect, which decreased the number of interconnected pores within the AASM system^58^. This reduction in pores mitigated the shrinkage stress induced by capillary effects, leading to a lower DS. Additionally, the addition of MK to GGBS introduced Al into the mix, thereby enhancing the crack resistance of the C(N)–A–S–H gel. A higher Al/Si ratio effectively reduced the sensitivity of AAMs to moisture loss during drying, contributing to a reduction in DS^20,59^.

### SEM analysis

**Figure 10:**
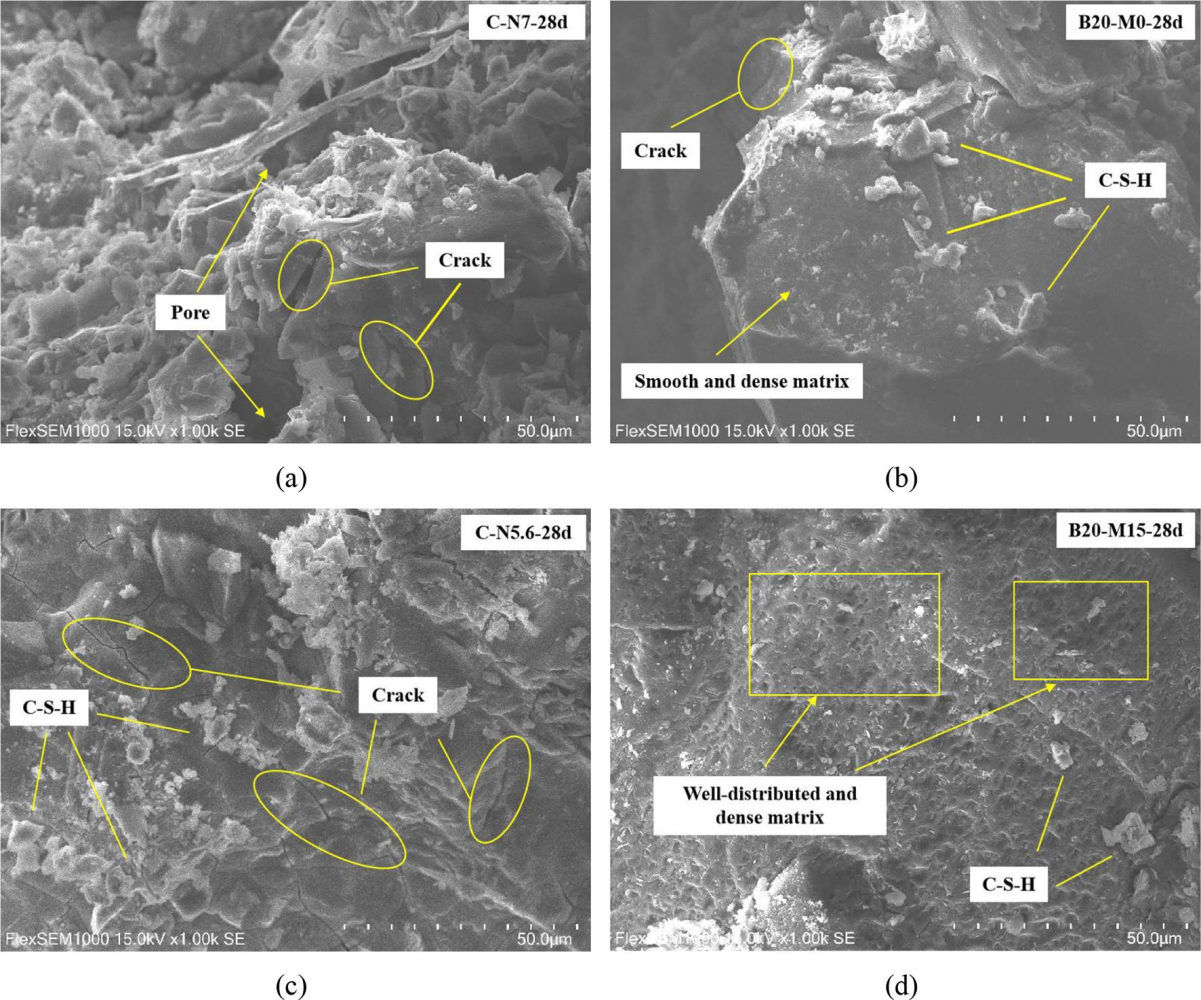
SEM micrographs of mortars at 28 d.

Figure 10a, c shows the SEM images of C-N7 and C-N5.6 at 28 d, respectively. The gel structure shown in Fig. 10a possessed a blocky and needle-like structure with significant local defects, which may be caused by a high alkali equivalent. The gel in Fig. 10c appeared more intact and was attached to some low-crystallinity hydrated calcium silicate gel, but numerous continuous microcracks were evident. Figure 10b, d shows the SEM images of the AAS (B20-M0) and AASM (B20-M15) mortars under the influence of the modified activator, respectively. As shown in Fig. 10b, the gel formed by hydration after the incorporation of borax was smoother and denser^52^, without noticeable micro-cracks, which microscopically explained that B20-M0 excited by the modified activator had better mechanical properties. The gel reaction degree shown in Fig. 10d was better than other three groups, and the surface was aggregated with flaky calcium silicate aggregates, which corresponded to the high reactivity of the MK. Additionally, the MK had a smaller particle size than GGBS, and the filling effect made the mortar structure denser.

### XRD analysis

**Figure 11:**
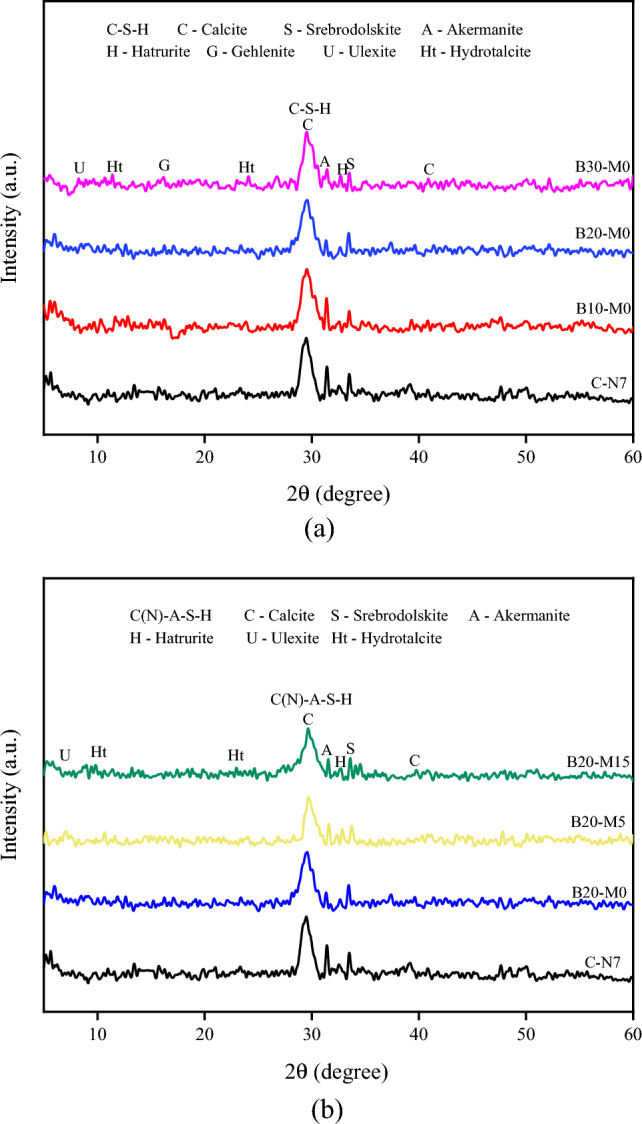
XRD patterns of hydration products of AAS and AASM pastes at 28 d: (**a**) with different borax contents and (**b**) with different MK contents.

Figure 11a, b presents the XRD patterns of the AAS and AASM pastes at 28 d, respectively. The main peak represented the calcite phase, and the phases on both sides were mainly calcium silicate hydrate. Ulexite (NaCaB_5_O_6_(OH)_6_(H_2_O)_5_) was present in the B10-M0, B20-M0, and B30-M0 samples. With the increase in the proportion of borax in the activator, the content of calcite and C–S–H gel remained relatively stable. The decreased intensity of diffraction peaks for akermanite and gehlenite, along with the emergence of ulexite diffraction peaks, suggested the involvement of borate ions in the formation of the gel network^37^. However, the excessive addition of borax reduced the amount of calcite generated, primarily due to the decrease in the proportion of sodium silicate in the activator. The reduction in alkali equivalent resulted in a decrease in the release of Ca^2+^ and Al^3+^ ions from GGBS, thereby impacting the strength of the mortar. This validated the analysis of the mortar's mechanical properties. With the increase in the substitution ratio of MK for GGBS, there was a higher content of Al_2_O_3_ and SiO_2_, leading to the formation of C(N)–A–S–H gel in the matrix^20,53,60^. However, no distinct diffraction peaks were observed, possibly due to potential overlap with the diffraction peaks of calcite^61^. Additionally, the formation of hydrotalcite is advantageous for the corrosion resistance of AAMs^62^.

### FTIR analysis

**Figure 12:**
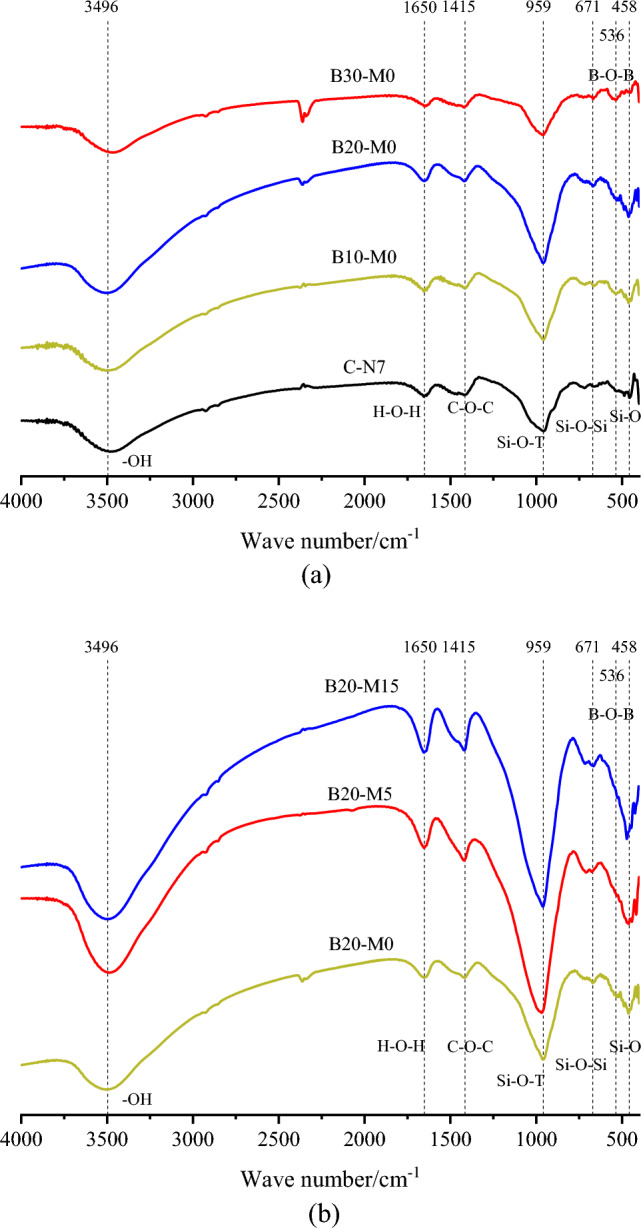
FTIR spectra of AAS and AASM pastes: (**a**) with different borax contents and (**b**) with different MK contents.

To elucidate the effects of borax and MK on the phase transition of AAMs samples, FTIR spectra analysis was performed on the bands of Si–O–T (T represents Si, Al or B), O–C–O, and H–O–H, which correspond to gels, calcium carbonate, and chemically bound water, respectively^63^. Figure 12a exhibits the effects of different borax levels. The spectral band near 1650 cm^−1^ represents the bending vibration of H–O–H^64^. As the increased of borax content, the peak of H–O–H band increased. This may be caused by the crystallization of borate compounds. The spectral band near 1415 cm^−1^ represents the symmetric tensile vibration of O–C–O^65^, which is associated with the carbonization of the sample. In all samples, there was a major absorption peak near 1109 cm^−1^, corresponding to the asymmetric tensile vibration of Si–O–T bonds caused by the dissolution of the silicate phase. The absorption peaks at 671 cm^−1^ and 536 cm^−1^ represent the symmetric tensile vibration of Si–O–Si, the bending vibration of B–O–B, respectively^37,66^. These peaks are associated with the formation of gel. With an increase in borax content to 20%, there was a significant enhancement in the spectral bands of Si–O–T and B–O–B, indicating that the internal polymerization reaction within the AAS mortar was strengthened following the use of borax-modified activator. This resulted in an increased quantity of the gel phase. This may be related to the involvement of B-O bonds in the composition of the gel network, and the resulting C–S(B)–H increases the degree of ploymerization of the gel^67^. Figure 12b exhibits the effects of different MK levels, and the peak of Si–O–T band increased with the increase in MK contents. This was attributed to increased levels of Si and Al in the mortar, resulting in additional C(N)–A–S–H gel formation. The increase of the peak of H–O–H and –OH bands indicated that the AASM mortar had a higher bound water than the AAS mortar, which was conducive to further mitigating shrinkage stress and reducing the DS of the AASM mortar.

## Conclusion

The effects of the borax-modified sodium silicate activator on the properties of the AAS mortar were investigated, and the optimal substitution level of borax was determined. Under the condition of an optimal borax replacement rate, the performance of the AASM mortar with different MK contents to replace GGBS were studied. The conclusions drawn from the test results are as follows.

- The fluidity of the AAS mortar initially increased and then decreased with higher anhydrous borax content in the activator, mainly due to the changes in the Na_2_O content and the consumption of free water. The increase in MK content led to a decrease in the fluidity of the AASM mortar, attributed to the smaller particle size and higher water absorption of MK compared to GGBS.
- The incorporation of an appropriate amount of borate enhanced the AAS mortar's mechanical properties by contributing [BO_4_] from borax dissolution, actively participating in the gel network with [SiO_4_] and [AlO_4_]. The addition of MK significantly enhanced the strength of the AASM mortar, attributed to MK's smaller specific surface area facilitating geopolymerization.
- The reduction in the DS of the AAS mortar was attributed to the decrease in alkali equivalent, reduction in mesopores, and the formation of borate compounds with high moisture retention properties.
- The incorporation of MK further reduced the DS of the AASM mortar. This was primarily attributed to MK's finer particle size and larger specific surface area than GGBS. These characteristics enhanced the interfacial bonding and compactness while exhibiting an ultrafine differentiation effect, reducing interconnected pores and mitigating shrinkage stress caused by water loss.
- The borax-modified activator reduced the DS of AAMs, while also reducing the dosage of sodium silicate activator. To broaden the application range of AAMs and promote environmental protection, it is recommended to investigate the effects of composite activators with boron-containing waste solutions on AAM's shrinkage and durability.

## Data Availability

Data will be made available on request. Data can be obtained from the corresponding author. (E-mail address: 2009028@aust.edu.cn).
